# Prospects and challenges for the genetic counsellor profession in the German-speaking countries: report of a workshop

**DOI:** 10.1515/medgen-2021-2055

**Published:** 2021-05-14

**Authors:** Gunda Schwaninger, Simone Heidemann, Wera Hofmann, Tamara Maurer, Katharina Mayerhanser, Joelle Ronez, Herdit Schüler, Katharina Steinmüller, Sabine Rudnik-Schöneborn, Johannes Zschocke

**Affiliations:** Institute of Human Genetics, Medical University of Innsbruck, Peter-Mayr-Straße 1, 6020Innsbruck, Austria; Institut für Tumorgenetik Nord, Kiel, Germany; genetikum, Stuttgart, Germany; Institute of Human Genetics, Technical University Munich, Munich, Germany; Institute of Human Genetics, Medical University Hannover, Hannover, Germany; Institute of Human Genetics, Universitätsklinikum Aachen, Aachen, Germany; Medizinisch Genetisches Zentrum (MGZ), Munich, Germany

**Keywords:** genetic counselling, genetic counsellor, genetics services, professional development, education

## Abstract

The genetic counsellor profession has not yet been established in the German-speaking countries. In 2019 the Medical University of Innsbruck inaugurated the first German-taught Master’s degree programme in Genetic and Genomic Counselling. In order to discuss prospects and challenges of the genetic counsellor profession in Germany, Austria and Switzerland (DACH region), the MSc programme team organized a two-day workshop with international speakers and medical geneticists from the DACH region. Day 1 was dedicated to the history, training and international profile of the genetic counsellor profession. Day 2 focused on four specific topics: (i) professional role, (ii) acceptance and job title, (iii) formal requirements and (iv) remuneration concepts for genetic counsellors in the DACH region. The workshop showed that the key factor for the successful implementation of the genetic counsellor profession is acceptance and trust within the medical genetics team. Genetic counsellors complement patient care in aspects that might be underserved considering the increasing demand of counselling in genomic medicine. Successful establishment of the genetic counsellor profession will entail the development of interprofessional teams under medical supervision and in the team of medical geneticists.

## Introduction

The enormous growth of genetic knowledge, rapid methodological advances and the fast-increasing implementation of genetic tests in medical care have sparked a surging demand for genetic information and counselling. The complexity of this need varies with the specific situation. Assistance in personal decisions on predictive tests that provide fundamental information on future health risks is more challenging than obtaining informed consent for a confirmatory test as part of the differential diagnostic work-up. Diverging setups of genetic services have been developed in different countries to ensure that valid decisions are made with regard to genetic investigations and that patients and their families gain maximum benefit – and suffer no harm – from these analyses.

Genetic counselling aims to facilitate informed decision-making and foster health-supporting behaviours of individuals with regard to genetic tests. It is a complex communication process that not only includes transmission of specialist information but also intends to assist individuals in a personal decision. In Germany and Austria, genetic counselling before and after relevant genetic tests is exclusively performed by medical doctors who must have a specialist qualification. Internationally, an additional healthcare profession, the MSc trained genetic counsellor (GC), has been introduced to support the work of medical geneticists [1]. GCs work in interprofessional genetics teams with medical and laboratory geneticists in building a strong foundation for genetic services [2]. They are part of the support process of patients and their families, especially after a genetic condition has been identified in the family [3].

**Figure 1:**
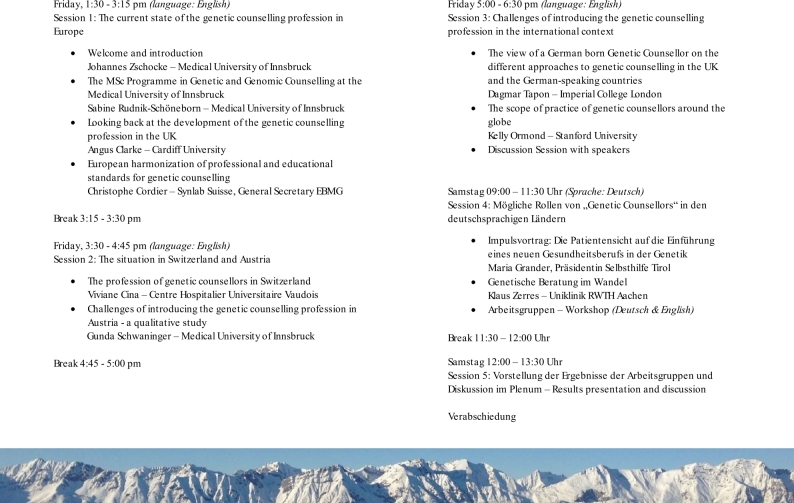
Programme of the two-day workshop on the ‘Role of Genetic Counsellors in the German-speaking countries’.

Following the international example, the Medical University of Innsbruck, in cooperation with the Health University of Applied Sciences Tyrol, has launched the first German-taught MSc programme in Genetic and Genomic Counselling in October 2019 with a first cohort of seven students. The programme team of the Innsbruck MSc course held an open workshop engaging medical geneticists from the DACH region (Germany, Austria and Switzerland) and international experts in the field of genetic counselling. This article is co-authored by the faculty and the first student cohort of the graduate programme. It presents an overview of the scope of practice of GCs in Europe and the USA and summarizes the opinions of the workshop participants on the possible professional role of GCs in the German-speaking countries.

## Workshop structure and participants

All German-language University Institutes of Human Genetics were invited to the workshop by personal announcement and repeated emails. Due to COVID-19 travel restrictions, the original date in June 2020 had to be moved to October 2020 and the workshop was held in an online format. The two days included 10 presentations by international speakers (Figure 1). Day 1 was dedicated to the history, training and international profile of the GC profession. It was accessed by an audience of about 50 participants including professionals and students from Austria, Germany, Italy, Sweden, Switzerland, the United Kingdom and the United States (28 from the DACH region, of which approximately half were medical geneticists). The focus of Day 2 were discussions on four specific topics: (1) the professional role of GCs in the DACH region, which tasks can be performed by GCs and which require full medical training; (2) conditions for the acceptance of GCs, choice of the professional title; (3) formal requirements that are necessary or desirable; and (4) remuneration concepts. Participants were divided into three breakout groups to discuss each topic, followed by a consensus discussion in the online plenum. Day 2 was attended by 32 active participants from the DACH region and the UK, of whom 17 were medical geneticists, 3 GCs, 7 genetic counselling students and 5 from other related professions.

**Figure 2:**
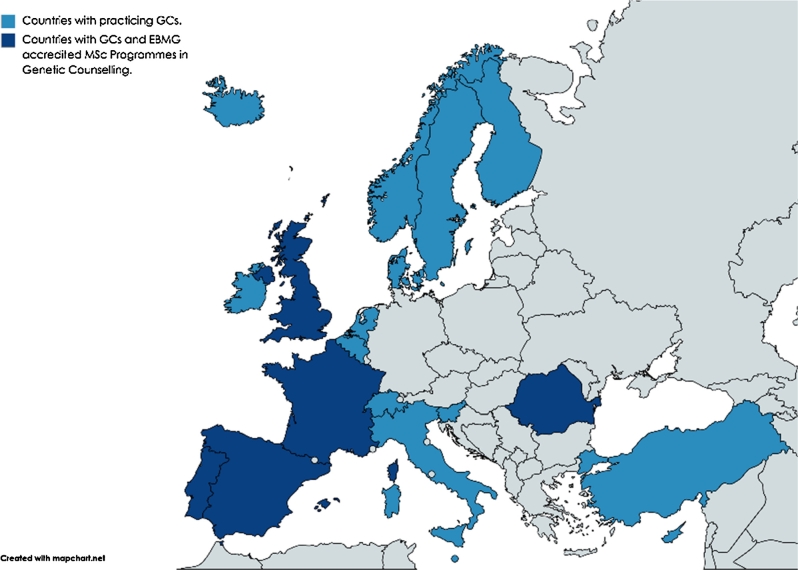
European countries with practicing GCs and EBMG accredited university programmes [4], [5].

## Background

### History of the genetic counselling profession and international status

The GC profession was established in the USA and dates back to 1947, when Sheldon Reed first defined genetic counselling ‘as a kind of genetic social work’ acknowledging the important psychosocial aspects of the role [6]. The first master-level graduate programme in genetic counselling was introduced at Sarah Lawrence College in New York in 1969; Canada followed with its first educational programme in 1980. To date about 50 university programmes are offered in North America. In 1973 and 1979, the US National Society of Genetic Counselors (NSGC) and the Canadian Association of Genetic Counsellors (CAGC) were founded [4], [5]. In Europe, the first graduate programme for genetic counselling was established at Manchester University in 1992 [7]. In the UK early GC roles developed from nursing and research, when individuals took on long-term relationships with families affected by genetic conditions to provide a continuing care setting. Today, the GC workforce with more than 200 registered GCs is working closely with medical geneticists and is a well-integrated part of the interprofessional genetics teams in the UK (Angus Clarke, Cardiff University, workshop report). In the last two decades the GC profession expanded to a total of 19 European states (Figure 2).

In most countries the GC profession developed primarily through GCs trained abroad or with other backgrounds like research or nursing, who were trained and employed in individual medical genetics centres. An exception is France, where the government perceived a need of non-physician professionals in medical genetics. This led to the government-induced establishment of a GC training programme at Aix-Marseille II University in 2004 and resulted in a strong legal basis from the onset of the profession [8]. A 2016 survey with 126 professionals working in genetics services showed that French GCs manage a wide range of cases independently but under the responsibility of medical geneticists. After more than a decade of practice the GC profession is well accepted by medical geneticists, and this mutual trust was reported to have been the crucial factor of success [9], [10]. The French part of Switzerland followed and today has a total of 11 GCs. The Association Suisse des Conseillers en Génétique (ASCG) was founded in 2016. Switzerland has recently revised its law on genetic investigations of humans (*Bundesgesetz über genetische Untersuchungen beim Menschen* [GUMG]) and states that genetic counselling has to be performed by a competent person (‘fachkundige Person’) [11]. The canton Vaud has established the first official regulation of GCs, and it is hoped that there will be an expansion of the profession to all Swiss cantons. Professional recognition and full acceptance of GC competences in Switzerland are major goals of the ASCG (Viviane Cina, Lausanne, Centre Hospitalier Universitaire Vaudois, workshop report).

In recent years the German Society for Human Genetics (GfH) has raised the option of introducing non-physician GCs also for Germany [12], [13]. In its third report, for the years 2016–2018, the German Commission on Genetic Diagnostics (Gendiagnostik Kommission [GEKO]) highlights the need to expand the currently limited capacities for genetic counselling in Germany. The introduction of non-physician GCs is explicitly proposed [14]. In 2019, the GfH and the Berufsverband der Deutschen Humangenetiker e. V. (BVDH) have submitted a request to the German Ministry of Health in which they propose the introduction of GCs to build a sustainable genetics work force at times of rising demand [15].

### Scope of practice, training and regulation of genetic counsellors

The scope of practice and the institutional framework for GCs differ between countries worldwide [4], [16], [17]. At the workshop, Kelly Ormond (Stanford University) presented results from a large-scale international survey on similarities and differences of GC practice across countries. Textbox 1 lists the most frequently reported core tasks by international respondents. More medical activities (e. g. performing dysmorphology exams, taking physical measurements, determining the presence of birth defects, ordering tests) were reported infrequently by GCs globally.

**Textbox 1:** Core tasks performed internationally by GCs (Kelly Ormond, Stanford University, workshop report)

– Review patient (and family) medical records
– Analyse pedigrees
– Integrate medical, laboratory and genetic information
– Identify family members at risk
– Educate clients and convey information about basic genetic concepts
– Explain possible testing options
– Facilitate informed decision-making and adaptation to genetic risk or conditions
– Explain outcomes and implications
– Discuss potential costs, risks, benefits, limitations and alternatives to testing
– Interpret clinical significance of test results
– Discuss test results and potential implications/limitations (e. g. sensitivity, specificity, residual risk)
– Recognize factors that may affect the counselling interaction (psychosocial assessment)

With the rapid development of genomic technologies, the traditional GC roles are changing. In the UK, with increasing demands on GC time, more attention is now paid to providing and explaining genetic information and less to the traditional counselling and care aspects of the GC profession (Angus Clarke, Cardiff University, workshop report). Nevertheless, a major focus of the GC work remains on the support of couples and families including the explanation of highly distressing, often uncertain genetic test results, support in the decision-making process and the discussion on how genetic tests can affect whole families. GCs also provide support after pregnancy loss and assistance in new pregnancies. Tasks that are strictly restricted to medical geneticists include diagnostic algorithms of genetic conditions, the physical examination, the evaluation of children with developmental delay and medical decisions on treatment (Dagmar Tapon, Imperial College London, workshop report).

With their combined training in genetics and psychosocial communication skills, GCs add to the quality of genetic services. In an era increasingly dominated by data-driven analyses and algorithmic decision-making, a profession that focuses on the counselling relationship with patients plays an essential role [18]. Studies provide evidence that active patient engagement can lead to increased compliance as patients are more likely to engage in health-promoting behaviour [19], [20]. Genetic counselling outcome studies have shown that genetic counselling by GCs leads to patient empowerment through increased knowledge, perceived personal control, positive health behaviours, improved risk perception accuracy and patient satisfaction. This is associated with a reduction of anxiety, cancer-related worry and decisional conflict [21], [22], [23]. GCs contribute to case management, fostering a person-centred approach and continuous support of whole families. They have been reported to be more accessible than medical doctors [18], [24].

**Figure 3:**
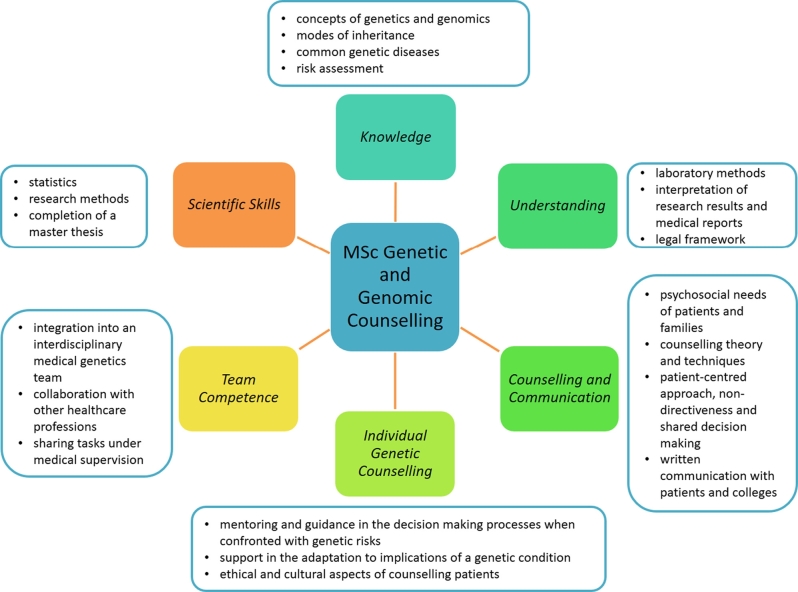
Major educational goals of the MSc graduate programme in Genetic and Genomic Counselling at the Medical University of Innsbruck.

In the last decade, Europe has seen major efforts to harmonize GC education, professional practice and registration in order to reach a broader acceptance and acknowledgement of GCs. The European Board of Medical Genetics (EBMG) developed a regulatory framework with competencies and standards for GCs practicing in Europe [25], [26]. Since 2013, the EBMG offers graduates with an accredited MSc degree and subsequent two-year professional experience in a medical genetics institution the option to register as a European certified GC (Christophe Cordier, Synlab Suisse, Secretary General EBMG, workshop report). This registration ensures Europe-wide professional recognition, international reciprocity of training and thus the opportunity to practice internationally [27].

The MSc graduate programme in Genetic and Genomic Counselling at the Medical University of Innsbruck is a five-semester part-time programme. It follows the EBMG core curriculum and consists of face-to-face and online teaching blocks and a 15-week practical placement at a medical genetics centre. The major educational goals are depicted in Figure 3. The curriculum focuses on (i) evidence-based genetics knowledge, (ii) communication and counselling skills and (iii) practical experience as key qualifications.

Prerequisite for enrolment is the successful completion of a relevant bachelor’s degree in a related biomedical science (e. g. biology, genetics), life science (e. g. psychology, healthcare, midwifery) or social science (e. g. communication science); work experience in the field of medical genetics and/or caretaking experience is desired. The application process includes a letter of motivation, a curriculum vitae, proficiency in the German and English Languages and an interview with the programme team. A new student cohort will be accepted in autumn 2021 (relevant information is available at www.gencouns.at).

### Results of the workshop discussions

On Day 2 of the workshop, participants were asked to join breakout groups with colleagues from different countries. Each group included representatives from all three German-speaking countries and discussed four topics that were introduced as questions. The results were subsequently presented and discussed in the virtual plenum. The conclusions of this process are summarized as follows:

1. *Which professional role should GCs play in Germany, Austria and German-speaking Switzerland? Which tasks can they take on, which tasks require medical specialist training?*

Participants agreed that the internationally developed core activities of GCs (Textbox 1) should also be suitable for GCs in the German-language context. GCs should be able to handle structured cases that do not require diagnostic decisions but focus on established diagnoses/risk constellations under medical oversight. There was clear consensus at the workshop that physicians who supervise GCs must have in-depth knowledge of human genetics in order to be able to assist in occasional critical situations. This implies full qualification as a specialist medical/human geneticist. Details of suitable and non-suitable tasks as summarized at the workshop are given in Textboxes 2 and 3.

2. *How can one create acceptance for the introduction of GCs in the German-speaking countries? What role does the job title play?*

It was generally agreed that acceptance of GCs within the genetics departments is the first and probably most crucial step for the profession to take [10]. This requires a clear definition of the different roles of medical geneticists and GCs in the provision of genetic services. In 2015, the UK Clinical Genetics Society published results from a workshop on the evolving role of medical geneticists, anticipating changes in the practice of the medical specialty [28]. With increasing demands of clinical and laboratory genetic diagnosis and the complex relationship between genotypes and phenotypes, the work focus of medical geneticists is expected to shift away from the traditional role of a ‘Genetischer Berater’ also in the German-language countries. As complementary members in the interdisciplinary team, GCs add flexibility to the tasks of the medical geneticist. Nevertheless, the experience from other countries with a recent introduction of the GC profession indicates that it takes time to reach general acceptance. An essential task is to make medical geneticists in the DACH region more knowledgeable of the scope of practice and the complementarity of the GC profession. It is hoped that familiarity with the profession will gradually develop as MSc graduates prove the added value GCs can bring to genetic services [24] that goes far beyond administrative support of medical geneticists, as GCs are often viewed as ‘genetic super-secretaries’. There will be a need for major advocacy efforts in the medical genetics community, professional associations, collaborating medical specialties and patient advocacy groups, as well as the public.

*What role does the professional title play?*

The term ‘genetic counselling’ refers to a process while ‘genetic counsellor’ is a professional title. The task of genetic counselling is provided by GCs as well as medical geneticists, but the process includes different tasks depending on who performs it. The participants at the workshop confirmed that in the German language, linguistic framing of the professional title is particularly sensitive. The German translation of genetic counselling – ‘genetische Beratung’ – is very tightly linked to the professional identity of medical geneticists [29]. The GfH has recognized that this is a disadvantage for the medical profession. Therefore, the GfH recommended to rename ‘Genetische Beratungsstellen’ into ‘Klinisch-Genetische Ambulanzen’ and to reserve the term ‘Genetische Beratung’ for the communicative part of the interaction that takes place during the genetic consultation [30]. Still, medical genetic specialists are frequently referred to as ‘Genetische Berater’. Using the verbatim German translation as the job title of a new healthcare profession in genetics would thus very likely hinder the acceptance of GCs. Different options were considered at the workshop. The recently coined term ‘Genetischer Beratungsassistent’ was deemed inadequate since it does not reflect the professional qualification of GCs and may cause confusion with the profession of ‘genetic counselling assistants’ recently introduced in the UK and the US for staff members without MSc degree who assist GCs by performing administrative tasks [16], [31]. The general consensus of the workshop participants was to introduce the new term ‘Genetischer Counsellor’ as a German adaptation of the English job title, in line with similar adapted terms such as ‘Manager’. This should secure international coherence and would reduce the risk of confusion with genetic counselling assistants and the role of medical geneticists.

3. *Which formal requirements are necessary or desirable? Is it currently possible to employ GCs under existing legal regulations in Germany, Austria and Switzerland?*

An MSc degree followed by a two-year traineeship in a medical genetics centre is the educational standard for internationally practicing GCs and necessary for certification as an EBMG registered GC. Students in Innsbruck are trained to fulfil all international requirements. Even if the scope of practice and the legal underpinning for the German-speaking countries is only developing, they are educated to be able to work in any European country.

**Textbox 2:** Tasks that GCs can take on under supervision of a medical geneticist

– Initial pre-assessment with medical geneticist
– Preparatory assessment of medical documents
– Individual and family history including pedigree analysis
– Genetic risk assessment, identification of family members at risk
– Clarification of the genetically relevant constellation and individual questions
– Re-assessment with medical geneticist if new aspects appear

Communication of genetic concepts, individual risks and genetic testing options

– Information about genetic concepts and inheritance patterns
– Discussion of genetic risks
– Information about genetic test principles and options

Decision support

– Clarification of individual patient preferences
– Facilitation of informed decisions
– Individual strategies for risk adaptation

Psychosocial assessment and support

– Psychosocial evaluation and supporte. g. in the case of a pregnancy, cancer disposition or life-changing diagnosis
– Space for difficult conversations
– Discussion of the individual meaning of genetic test results
– Expansion in counselling aspects that are underserved
– Shared consultation with the medical geneticist to balance different aspects
– Contact support with self-help groups

Post-test processing

– Written communications for doctors as well as patients and their families
– Joint case review by GC and medical geneticist
– Case discussion in the interdisciplinary team

**Textbox 3:** Tasks that are not suitable for GCs

– Evaluation of developmental disorders/syndromes
– Physical examination or dysmorphological assessment
– Phenotype analysis
– Discussion of differential diagnoses
– Choice of the genetic testing strategies
– Referral to other medical disciplines
– Discussion of treatment options
– Complex consultations

**Textbox 4:** Elements of creating acceptance for the introduction of GCs

– Interaction with GCs – employment in medical genetics centres or practices (in a legally correct setting)
– Precise definition of the professional profile and scope of practice of GCs
– Clarification of the remuneration of GCs
– Clarification of how the medical genetics team benefits from employing GCs
– Resolution of competence disputes with other specialists/other professional groups
– Fostering of the clinical self-understanding and profile of medical geneticists
– Increase of public awareness and policy engagement

According to the German *Gendiagnostikgesetz* (GenDG) it is possible to include non-physician experts for advice with the consent of the person concerned (§ 10 (3) GenDG). Similarly, the Austrian *Gentechnikgesetz* (GTG) requires counselling by a genetically trained medical specialist but in relevant circumstances additional non-medical counselling with a psychologist/psychotherapist or social worker must be offered (§ 69 (4) GTG) [32]. This law was implemented without GCs in mind but makes it clear that it is not only possible but sometimes advisable to include other professionals in genetic consultations. The corresponding Swiss *Bundesgesetz über genetische Untersuchungen beim Menschen* (GUMG) in its 2018 revision (the law has been passed but not yet implemented) goes further by stipulating that a physician who orders a genetic analysis must offer or organize genetic counselling, but this can be carried out by a competent person (‘fachkundige Person’) (§ 21 (2) GUMG) [11].

There was agreement among the workshop participants that the process of genetic counselling must be under the supervision of a board certified and experienced geneticist who is also responsible for ordering genetic analyses and obtaining informed consent. Nevertheless, even under the current restrictive laws in Germany and Austria it is thought to be legally possible to involve qualified GCs in the counselling process under the oversight of specialized medical geneticists. The revised Swiss law already allows genetic counselling to be performed by qualified GCs as a ‘competent person’. The workshop participants agreed that full training at MSc level and subsequent work experience as stipulated by the EBMG should be the minimum requirement for professionals who are directly involved with patients in the genetic counselling process. Students entering the MSc programme should have prior experience in dealing with ‘vulnerable people’ and/or work experience in a genetics centre. Full acceptance as a licenced healthcare profession (Gesundheitsberuf) should be aimed for in all countries of the DACH region.

4. *Which remuneration concepts are suitable for GCs in relation to other non-medical professions?*

As posts for GCs are created, it has to be evaluated where to place them in existing salary structures. The UK pays GCs according to the ‘Agenda for Change’ band pay scale [33]. In the US GC payment varies as local living costs differ substantially and GCs have more diverse roles. The workshop groups mentioned several other healthcare professions that may be perceived as comparable, e. g. psychotherapists, occupational therapists or highly qualified laboratory technicians. In general, salaries are based on qualification, academic degree, professional experience and job responsibilities. As such, GCs are not normally expected to reach the remuneration levels of clinical laboratory geneticists (‘Fachhumangenetiker’), who usually have a PhD degree and a minimum of five years’ postdoc training.

Several participating medical geneticists from Germany work in community-based practices not linked to a (university) hospital. As genetic counselling is a genuine outpatient activity, a crucial question concerns the remuneration of GC activities by health/social security services and insurance companies. The introduction of adequate refund numbers for billing, both in institutional and private settings, is a challenge for the future.

## Conclusions

So far GCs represent an unfamiliar group of professionals in genetic services in the German-speaking countries. Internationally, GCs join and support medical genetics teams with their special communication skills. They support the decision-making process of individuals who are affected by a genetic condition or have a specific genetic risk, grant continuity of care in the follow-up of distressing and uncertain situations, foster communication about genetic conditions within families, and act as patient advocates within the medical genetics team but also in the liaison with patient advocacy groups. The key factor for the successful implementation of this novel profession is to achieve their integration into interprofessional teams led by medical geneticists. GCs are not striving to rival or challenge the existing medical and healthcare professions but to contribute to patient care especially in the counselling aspects that might otherwise be underserved. This is seen as a complementary role, in addition to current provision, particularly when considering the rapidly increasing demand for counselling in the context of genomic medicine.

The workshop confirms study results that the semantic framing of the new profession will play a major role in the acceptance of GCs in the German-language setting, as the term ‘Genetische Beratung’ is tightly linked to the professional identity of medical geneticists in Germany, Austria and Switzerland. The scope of GC practice must be carefully defined and explained, and the professional title has to be carefully chosen to avoid possible conflicts. The development and introduction of a novel healthcare profession in medical genetics is a long-term process that has only just been initiated. There is a need for a broad and open discussion of diverse opinions about the future role of GCs in the German-speaking countries.
